# The Cancer Stem Cell in Hepatocellular Carcinoma

**DOI:** 10.3390/cancers12030684

**Published:** 2020-03-14

**Authors:** Lucas-Alexander Schulte, Juan Carlos López-Gil, Bruno Sainz, Patrick C. Hermann

**Affiliations:** 1 Department of Internal Medicine I, Ulm University, 89081 Ulm, Germany; lucas-alexander.schulte@uniklinik-ulm.de; 2 Department of Biochemistry, Universidad Autonoma de Madrid (UAM), 28029 Madrid, Spain; juancarlosbio95@gmail.com; 3 Department of Cancer Biology, Instituto de Investigaciones Biomedicas “Alberto Sols” (IIBM), CSIC-UAM, 28029 Madrid, Spain; 4 Chronic Diseases and Cancer, Area 3-Instituto Ramon y Cajal de Investigacion Sanitaria (IRYCIS), 28029 Madrid, Spain

**Keywords:** cancer stem cells, hepatocellular carcinoma, liver cancer

## Abstract

The recognition of intra-tumoral cellular heterogeneity has given way to the concept of the cancer stem cell (CSC). According to this concept, CSCs are able to self-renew and differentiate into all of the cancer cell lineages present within the tumor, placing the CSC at the top of a hierarchical tree. The observation that these cells—in contrast to bulk tumor cells—are able to exclusively initiate new tumors, initiate metastatic spread and resist chemotherapy implies that CSCs are solely responsible for tumor recurrence and should be therapeutically targeted. Toward this end, dissecting and understanding the biology of CSCs should translate into new clinical therapeutic approaches. In this article, we review the CSC concept in cancer, with a special focus on hepatocellular carcinoma.

## 1. Introduction

In solid tumor oncology, there are only few examples of medical treatments that are curative or that achieve sustained and long-term remission. In general, most patients retain at least some tumor burden and/or suffer relapse after initial treatment response. Several hypotheses have tried to provide an explanation for this phenomenon. The clonal evolution model refers to a model in which a therapy is administered at doses that cause tolerable, non-permanent damage to healthy tissues, causing only moderate side effects, whereas tumor cells, which are more susceptible to therapy, are targeted and eliminated [1]. Based on pure probability, however, some cancer cell clones will possess resistance to therapy due to additional acquired mutations or microenvironmental cues that will facilitate their survival, giving rise to a new clonal tumor that can grow despite continued treatment. In the clonal evolution model, heterogeneity within each tumor is a result of randomly acquired (and subsequently selected) alterations, and every tumor cell equally contributes to the fate of the tumor. Furthermore, all cancer cells have tumor-initiating capacity, and those that survive therapy will drive relapse.

In a way, the clonal evolution model fits the Darwinian concept of “survival of the fittest.” Those cells that adapt to new challenges confer a selective advantage to their progeny. This can lead to therapy-induced selection of a dominant clone with new biological features, and to the loss of those cells that were “addicted” to the previous molecular target, as has been observed with anti-EGFR (epithelial growth factor receptor) therapy in colorectal carcinoma [2]. While clinical observations such as acquired drug resistance or tumor relapse can be explained by the clonal evolution model, evidence to the contrary exists. Most compelling are studies showing that tumorigenic activity is restricted to certain cancer cell populations. That is, the majority of “bulk” tumor cells either fail altogether to generate tumors in xenotransplantation models, or exponentially higher numbers of cells have to be transplanted in order to generate a tumor [3]. At the same time, distinct subpopulations of cancer cells with specific surface marker expression profiles are exceptionally tumorigenic and give rise to fully-fledged tumors after xenotransplantation of extremely low numbers of cells. This phenomenon was first observed in acute myeloid leukemia (AML) in 1994 by John Dick and colleagues [4]. In this study, the authors demonstrated that CD34+/CD38− AML cells had significantly higher tumorigenic potential in SCID mice than the CD34+/CD38+ or CD34− fractions. This discovery of intra-tumoral heterogeneity and tumor hierarchy led to the advent of the cancer stem cell (CSC) concept, with a CSC being a tumor cell that can self-renew, differentiate into all tumor cell lineages, exhibit exclusive tumor-initiating capacity and possess high chemotherapeutic resistance [5]. This model places the CSC at the apex of the tumor hierarchy, undergoing asymmetric and symmetric divisions, the latter ensuring perpetual self-renewal and maintenance of the CSC sub-population. Likewise, the model proposes that CSCs are exclusively able to intravasate into the circulation, survive and successfully metastasize to distant locations. Since metastasis and tumor relapse dictate overall survival, targeting CSCs provides new hope for improving long-term survival.

Hepatocellular carcinoma (HCC) is a frequent and prognostically poor malignancy of the liver. Currently, HCC represents the fourth leading cause of cancer-related death worldwide and the sixth most commonly diagnosed cancer [6]. HCC is typically divided into two molecular subgroups: (1) proliferation class, which is poorly differentiated, expresses high alpha-fetoprotein (AFP) levels and is commonly associated with hepatitis B virus (HBV) infection; and (2) non-proliferation class, which is more commonly associated with alcohol-related HCC or hepatitis C virus (HCV) infection [7]. Treatment of HCC is hampered by several factors: (1) the tumor is highly resistant to classical chemotherapy and is usually detected at late stages with no hope for complete surgical resection, and perhaps most importantly, (2) HCC typically develops in critically damaged cirrhotic livers that have suffered years of active hepatitis or toxin intake, and therefore, patient tolerance to therapy is severely limited. In the following article, we will summarize the role of CSCs in cancer, and provide an overview of the current knowledge of CSCs in HCC.

## 2. Cancer Stem Cells and Their Role in Cancer

### 2.1. Cancer Stem Cells

#### 2.1.1. CSCs and Their Clinical Relevance

The first evidence for the existence of CSCs came from studies in AML in 1994 by Dick and colleagues [4], who provided the first proof of a hierarchical architecture in a human cancer. By xenotransplanting AML cells into severe combined immunodeficient (SCID) mice, the authors found profound inter-patient differences in the cell numbers needed for leukemia induction. Specifically, after using FACS (sorting) on AML cells, the leukemia-inducing cells were exclusively found in a subset of CD34+/CD38− cells. These cells have been discovered in many other malignancies, such as pancreatic, breast, glioma, prostate, lung and liver carcinomas [8,9,10,11,12,13,14,15], and are referred to as CSCs, tumor-initiating cells (TICs) or cancer stem-like cells [16]. Importantly, throughout the last two decades, many groups have demonstrated that these sub-populations of cells exhibit inherent plasticity and dormancy, mimicking many of the characteristics of somatic stem cells [5]. Importantly, similarly to tissue-resident stem cells, the differentiation of CSCs into other tumor cells is not unidirectional. Meaning, a tumor’s CSC pool can be replenished by de-differentiation of “bulk cells” via a process referred to as transdifferentiation. This concept was experimentally shown in two studies from 2017. First, de Sousa e Melo et al. used mouse-derived colon cancer organoid cultures (Apc^min^; Kras^G12V^; p53^CRIPSR^; SMAD4^CRIPSR^) that expressed EGFP (enhanced green fluorescent protein) and the diphtheria-toxin (DT) receptor (DTR) under the control of the leucine-rich repeat-containing G-protein coupled receptor 5 (LGR5) promoter to initiate tumors in recipient mice [17]. The authors showed that DT treatment inhibited tumor growth concomitant with loss of the EGFP+/LGR5+ cells. Shimokawa et al. targeted the LGR5+ CSC population in organoid cultures of human colorectal cancer using an inducible suicide-gene caspase 9 (iCasp9) approach [18], and demonstrated that upon iCasp9 induction, organoid-derived xenograft growth in vivo was significantly reduced. Interestingly, upon removal of DT or the inducer, tumors regrew and the LGR5+ CSC population reemerged, indicating replenishment of the CSC pool from LGR5-negative cells. However, such transdifferentiation was not observed by Chen et al. in a mouse model of glioma, where the authors labeled glioblastoma cells using a nestin-ΔTK-IRES-GFP transgene. Upon treatment with ganciclovir (which specifically eliminates herpes simplex virus type-1 thymidine kinase (TK)-expressing cells) and temozolomide, tumor development was abrogated [19]; however, no replenishment of the CSC pool from other glioblastoma cells was observed.

The clinical relevance of the CSC model is based on the observation that the resistance of CSCs to chemotherapy and radiation makes them responsible for the relapse of malignant diseases [20]. Likewise, CSCs can undergo epithelial-to-mesenchymal transition (EMT), invade, circulate in the blood stream and extravasate at distant sites to form metastatic lesions [9,21,22,23,24,25]. Thus, from a therapeutic perspective, long-term effective anti-cancer therapies must also eliminate these CSCs, and much effort has been invested in defining and understanding the mechanisms underlying the aforementioned key properties of CSC biology. CSCs seem to enter a more dormant state with reduced proliferative activity. In this state of G0-arrest, cells resist chemotherapy and persist, sometimes for years, eventually causing relapse [26]. Furthermore, CSCs exhibit increased DNA repair and reduced apoptosis compared to bulk tumor cells [27,28,29,30], and strongly express drug efflux transporters, such as multi-drug resistant (MDR) proteins, a feature that can also be used to identify and isolate these cells [31]. Perhaps equally important with respect to the maintenance and protection of CSCs is the niche, a location within the tumor microenvironment (TME) that “nurses” CSCs by providing anti-apoptotic, stemness-maintaining factors and matrix components. This special TME is believed to not only help CSCs resist therapeutic efforts but also to play an important role in the trans-differentiation of non-CSCs into CSCs. 

#### 2.1.2. CSC Identification and Markers

Two hallmarks of CSCs are (1) their ability to re-establish tumors with the same molecular and phenotypic heterogeneity (and perhaps subtype) as the original tumor (differentiation), and (2) the maintenance of the CSC pool via unlimited self-renewal, ensuring that the CSC as the source of tumor replenishment is never lost. These two properties are typically validated in (xeno-)transplantation models where putative CSCs are implanted in mice and tumor formation is assessed, and in subsequent serial transplantation models to asses unlimited self-renewal. A large number of cellular markers have been used to identify and experimentally validate CSCs, including CD133, LGR5, CD44, Aldehyde dehydrogenase 1 (ALDH1), Mucin 1, ATP-binding cassette super-family G member 2 (ABCG2) and many more. However, it is important to note that to date no universal CSC marker or combination of markers have been identified/established. While the function of some of these markers in CSC biology is unknown, CSC-specific functions for many of these markers have been described. ALDH1 is one of the aldehyde-dehydrogenase isoforms and is overexpressed in many CSCs, clearing toxic aldehydes and lowering oxidative stress. Additionally, ALDH1 has been shown to confer a survival advantage to CSCs of many tumor entities, and its expression is relevant for prognosis [32,33]. CD133 (prominin-1) is a commonly used CSC marker. It is a lipid-raft bound transmembrane protein, which is involved in the activation of EMT-promoting signals, such as autocrine interleukin (IL) IL-1 and NFκB signaling [34,35,36]. It has been used successfully to identify CSCs in pancreatic [9] and breast cancer [37], hepatocellular carcinoma [38] and glioblastomas [15]. Several ATP-binding-cassette (ABC) proteins have also been identified as CSC markers. These MDR proteins are themselves upregulated by stemness-associated signaling pathways, such as Wnt, and act as active efflux pumps for several substances, including anti-cancer drugs, and are therefore functionally important [39]. Furthermore, ABC proteins such as ABCG2 are the basis for functional assays to enrich for CSCs. Staining with the DNA binding dye Hoechst-33342 identifies CSCs with high expression and activity of ABCG2, as only these cells are capable of depleting this intracellular stain, while non-CSCs will retain the dye. These non-Hoechst-retaining cells can be identified in cytometry plots as a “side population” [26]. Interestingly, ABCG2 was also shown by Miranda-Lorenzo et al. to promote the accumulation of riboflavin in cytoplasmic vesicles. This accumulation of riboflavin results in autofluorescence, which has been used successfully to enrich for CSCs in pancreatic, colorectal and liver cancers [40].

#### 2.1.3. The CSC Niche, the Tumor Microenvironment and Metastasis

While little is known about the CSC “niche,” like somatic stem cells, CSCs are likely to reside within a supportive environment that regulates their stemness [41]. The CSC niche is formed by the interplay of different cells and factors, which ensures the generation of a proper environment to maintain the CSC population. While the niche is a changing entity that undergoes significant variations depending on the location of the CSC(s) within the TME and the exact moment in time during tumor evolution, an assortment of common factors predominates throughout the life of the CSC niche. Fibroblasts, mesenchymal stem cells and immune cells are the most important support cells found in the CSC niche, although other cell types (e.g., endothelial cells) also contribute to the niche. These cells secrete factors that can modulate the fate of the CSCs, and CSCs will produce factors to adapt the niche to their needs [42,43]. In the end, a bidirectional and dynamic environment is formed between CSCs and the cells of the niche, and disrupting this environment could be detrimental for CSCs. Fibroblasts, a key structural cell type in many tissues, exert essential repair functions following tissue injury. In cancer, however, fibroblasts can become cancer-associated fibroblasts (CAFs), which are characterized by rapid proliferation and production of tumor-promoting factors (e.g., VEGF (vascular endothelial growth factor), SDF1 (stroma-derived factor 1), HGF (hepatocyte growth factor) or CXCLs (Chemokine [C-X-C Motif] Ligands)) that enhance tumor growth and progression [44]. The communication circuit between CSCs and CAFs is essential for maintaining stemness and is mediated by the activation of signaling pathways such as STAT-3-NF-κB [45], NOTCH [46] or Wnt [22,43]. This interplay can also promote migration, expansion and de-differentiation of bulk tumor cells into CSCs. The roles of CAFs and CSCs were recently reviewed in Alguacil-Núñez et al. in 2018 [47]. 

Mesenchymal stem cells (MSCs) are cells with the ability to differentiate into structural lineages (osteoblasts, adipocytes and chondrocytes) and have been isolated from a wide variety of organs and tissues [48]. Bone marrow-derived MSCs are recruited to the TME, providing the CSC niche with a combination of secreted cytokines that modulate the stroma [49], the immune system [50] and the MSCs themselves [51]. This facilitates CSC maintenance, but also chemoresistance within the TME [52].

Finally, the niche is also characterized by the infiltration of anti-inflammatory/pro-tumoral immune cells. Anti-inflammatory cells will be attracted to deal with the effect of pro-inflammatory innate immune cells (e.g., M1 macrophages and natural killer cells) and cytotoxic T cells. Tumor-associated macrophages (TAMs) and myeloid-derived suppressor cells (MDSCs) [53] enhance stemness and favor a pro-tumoral and pro-metastatic state by releasing cytokines (e.g., IL-6) that activate STAT3, ultimately inducing several stem-related pathways, EMT, and the expression of CSC markers, such as CD133 [54]. Underscoring the significance of the niche for the maintenance of CSCs, TAM depletion leads to a reduction in CSC numbers, tumor size and metastasis [55], while MDSC depletion increases the effect of cytotoxic T cells and reduces tumor growth [56].

Metastasis depends on several steps. First, a tumor cell has to lose its intercellular adhesions (via EMT) and invade the surrounding tissue, migrate to nearby lymphatic or blood vessels, enter the circulation, survive splenic and intravasal immune surveillance, stop its travel in a suitable target tissue, extravasate and form metastatic lesions (via reversed EMT; i.e., mesenchymal-to-epithelial transition (MET)). Increasing evidence suggests that the TME activates pro-metastatic signals in CSCs via factors secreted by the niche, or by other factors that affect the CSC state, such as hypoxia, acidic pH or glucose deprivation. VEGF is a pro-angiogenic transmitter with multiple functions in tumor biology. Intra-tumoral hypoxia is a key driver of VEGF production, and CSCs can produce VEGF [57]. Together, hypoxia and VEGF lead to the formation of atypical vessels with increased permeability. Furthermore, VEGF can induce EMT in CSCs, activating the circuitry needed for CSCs to lose their intercellular adhesions and enter nearby vessels [58,59,60,61,62,63]. Indeed, anti-VEGF treatments have been successfully used in AFP^high^ HCC patients [64], but their specific efficacy against CSCs is unclear [65,66]. The latter is likely due to CSCs being able to “hijack” angiogenesis in vasculogenic mimicry [67]; that is, CSCs forming the endothelium in tumor-associated vessels [68,69,70,71].

Once in the blood stream, CSCs have to overcome new obstacles. Only about 1 out of 500 CSCs is able to survive in circulation [72], and elaborate mechanisms are in place to protect CSCs from elimination by the immune system [73]. For example, CSCs decrease major histocompatibility complex I (MHC-I) expression and antigen presentation, evading immune cell attack via the expression of PD-group ligands and instigating bystander cells to create an immunosuppressive milieu. Furthermore, CSCs can shield themselves by forming adducts with transforming growth factor-beta (TGF-β)-producing platelets and fibroblasts, and they can protect their micro-milieu by forming spheres in the bloodstream [73,74,75,76,77,78,79,80,81,82]. To successfully form distant metastases, the metastatic site must provide the appropriate milieu necessary to support all essential CSC properties. Exosomes or exosomal-like extracellular vesicles (ECV) are small membrane-protected bodies that can carry bioactive compounds, such as micro-RNAs and proteins, to cells at distant sites. In cancer, it has been shown that these ECVs are responsible for the formation of pre-metastatic CSC niches in distant organs, where they adhere by site-specific integrin interactions. These pre-metastatic niches are required for the successful seeding of CSCs and are built with the collaboration and support of ECV-educated bone marrow-derived cells and macrophages that produce vascular structures and promote the activation of stemness-related pathways, such as Wnt, Notch or Src signaling [83,84,85,86,87,88,89,90]. Once at the metastatic site, CSCs are thought to attach to the nearby endothelium by integrin- and lectin-mediated cell adhesion molecules and start proliferating in the capillaries to which they adhere, followed by invasion [91,92].

## 3. CSCs and Their Role in Hepatocellular Carcinoma

### 3.1. Stem Cells in the Normal Liver 

Compared to other organs, the liver has the unique ability of extensive regeneration after damage-induced tissue loss. In fact, less than 50% of the original parenchyma is sufficient to restore the original liver mass [93]. Experiments have proven the liver to be capable of multiple cycles of self-renewal, raising hopes for regenerative therapeutic approaches [94,95,96,97]. Depending on the severity of the liver damage and possible replication restrictions of the remaining hepatocytes, several patterns of repair have been observed [98,99,100]. Partial hepatectomy prompts a strong growth signal to the remaining liver tissue, leading to the proliferation of hepatocytes and subsequent regeneration of the liver mass. In experimental settings, hepatocytes are able to divide more than 60 times without reaching replicative senescence or undergoing obvious functional loss [94]. However, other models of extensive liver damage show a reduced proliferative capacity of hepatocytes. In this case a distinct cell population residing in the periportal area near the smallest bile ducts starts to proliferate and to differentiate into hepatocytes and cholangiocytes in a “ductular reaction”. Many observations support this scenario, but there is also contradictory evidence from lineage-tracing experiments [101]. While the primordial hepatic stem cell responsible for cellular repopulation has not been definitively discovered to date, a distinct liver-resident “oval cell” most likely represents the bipotent progeny of these stem cells and proliferates to rebuild the lost parenchyma in mice [102]. Marker profiling shows heterogeneity amongst oval cells suggesting different lineages, but a common marker profile combines markers of biliary, hepatoblastic and hematopoetic progenitor cells [103,104,105]. This implicitly strict model of the potency of single cells, however, is contradicted by other studies showing that hepatocytes can transdifferentiate into other lineages without genetic manipulation, proving the bipotency of hepatocytes [106,107]. Although Sox9 lineage tracing of oval cells showed that these cells are bipotent in organoid cultures, they only marginally contribute to liver regeneration, even in the typical “ductular reaction” models [108]. Therefore, it could be hypothesized that the repopulative relevance of oval cells is not so much due to bipotency, but rather due to their resistance to hepatotoxic agents as a consequence of their less differentiated state/relative quiescence that prevents them from metabolizing hepatotoxins [109].

The gold standard to demonstrate cellular inheritance or cell fate is lineage tracing. In most cases, a cell type-specific promotor driving the expression of the Cre-recombinase and a loxP-STOP-loxP flanked reporter construct are inserted into the germline DNA. Thus, the (fluorescent) reporter is expressed when the cell type-specific promotor becomes transcriptionally active and Cre is expressed, leading to excision of the stop cassette and the permanent expression of the reporter construct in the cell and all its progeny. Models in which a hepatocyte has been shown to be the cell responsible for liver regeneration are supported by many transplantation and lineage tracing experiments [94,95,96,97,110], but there is also evidence that other cell populations inside and outside the liver are capable of liver regeneration [111,112,113,114,115]. The bone marrow is linked to hepatic stem cell pools in several ways. Oval cells exhibit markers suggestive of a bone marrow descendance [116], and hematopoiesis moves from the liver and spleen to the bone marrow during ageing [117]. Accordingly, it was shown in allogenic bone marrow transplant models that liver repopulation could ultimately stem from the grafted marrow [118]. While this seems not to have major relevance in liver regeneration following injury [119], the role of the bone marrow in HCC carcinogenesis has yet to be determined. MSCs originate from multiple tissues, including bone marrow or adipose tissue, and represent another possible source of hepatic progenitor cells [120], since studies have shown MSCs to be capable of engrafting in liver repopulation models [121,122]. 

The cellular compartment giving rise to the tumor-initiating cell in HCC is under intense debate, and either progenitor/stem cells or differentiated liver cells could be responsible. Experimental evidence indicates a model where every cell can be a key player [123]. There is profound inter-tumoral heterogeneity in HCC with respect to genetic profiles, allowing for the discrimination of at least two genetic/molecular subgroups, as described above [7,124], and transcriptional profiling can distinguish different prognostic subgroups. Interestingly, a subtype with a progenitor cell-like expression pattern has been identified and seems to be associated with activation of AP-1 transcription factors and poor prognosis [125]. Likewise, RNA and protein expression levels of Epithelial cell adhesion molecule (EpCAM) and AFP could define prognostically distinct subgroups of HCC, and EpCAM expression was associated with a progenitor gene expression signature, including tyrosine-protein kinase KIT (c-Kit), Wnt activity and CK19 expression [126]. In turn, a CK19 expression signature correlates with prognosis [127].

Several lines of evidence indicate that hepatic progenitor cells (HPCs) could represent the ancestors of the TIC in HCC. Embryonic liver fodrin (ELF) is a downstream target of TGF-β/SMAD signaling that is expressed in a stem cell-like subpopulation of the liver, indicating a possible “stem” cell origin. Interestingly, heterozygous ELF knock-out mice spontaneously develop HCC, regulated by hampered TGF-β signaling [128]. In another model, Wu et al. subjected an HPC-like cell line (WB-F344) to long-term in vitro treatment with TGF-β, resulting in an AKT-dependent enhanced tumorigenic potential in NOD-SCID mice [129], and suggesting HPCs to be the source of TICs in HCC. Genetically engineered fetal murine hepatoblasts (i.e., progenitor cells) harboring alterations inactivating p53 and activating several oncogenes (*c-myc*, activated *Akt* (*Akt1*) or oncogenic *Ras* (*H-Ras^V12^*)) formed tumors in preconditioned wild-type recipient livers [130]. Furthermore, attenuation of the developmental pathways Hippo and Neurofibromatosis type 2 (which have been associated with HCC) led to HPC expansion and tumor formation [131,132]. In β-catenin-stabilized mouse models, only HPCs can generate tumors, while hepatocytes need further genetic alterations to form malignant liver tumors [133,134]. Finally, restricting liver cell survival by epigenetic induction of G2-arrest combined with STAT3-activation leads to HCC formation with HPC-like features [135].

While there is significant evidence to support HPCs as the “cell of origin” in HCC, hepatocytes have also been shown to be responsible for HCC development. Lineage-tracing models revealed that in certain HCC models, tumors are derived from hepatocytes and not from HPCs. Using Hepatocyte nuclear factor -1beta (HNF-1β) as an HPC marker, no contribution to genetically or chemically-induced HCC could be attributed to HPCs [101]. In another hepatocyte tracing model, nearly all chemically or genetically induced HCCs were the progeny of mature hepatocytes [136,137,138]. Recently, a self-maintaining pericentral group of LGR5+ hepatocytes was shown to be highly susceptible to hepatocarcinogenesis, and was determined to be primarily responsible for tumor development in diethylnitrosamin (DEN)-induced HCC [110]. LGR5 regulates chemoresistance via Wnt potentiation, p53 suppression and EMT induction in HCC, all of which are typical characteristics of CSCs [139,140]. Furthermore, LGR5 is an established CSC marker in colorectal cancer [18,141]. These observations indicate that in HCC, the mechanism of CSC/TIC generation may be the induction of stem cell traits rather than cellular inheritance. This scenario is further supported by the observation that Nestin expression following p53 loss is associated with the dedifferentiation of mature hepatocytes into progenitor-like cells in hepatocarcinogenesis, a process that is mediated by lineage-specific mutations that target Wnt signaling [142].

### 3.2. Identification of CSCs in HCC

**Figure 1:**
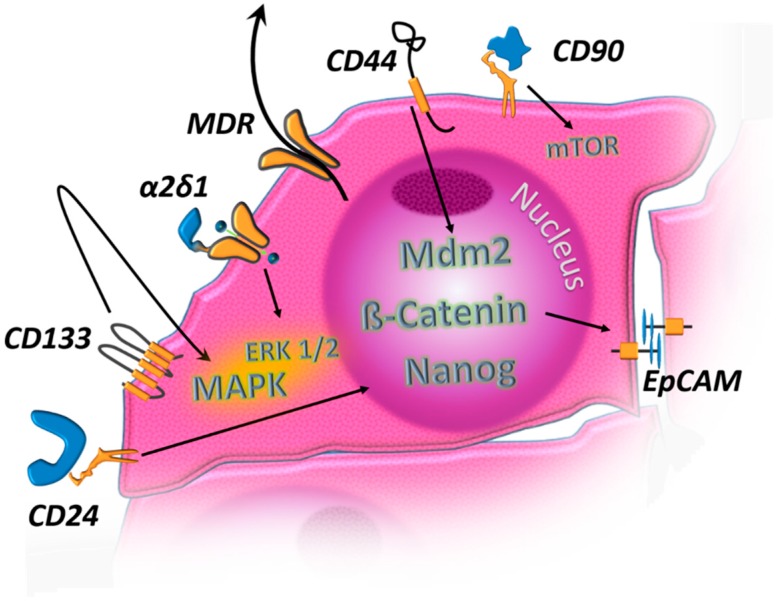
Established markers for cancer stem cells in hepatocellular carcinoma (HCC) and possible functions. MDR: multidrug resistance protein, ATP-dependent substrate export; α2δ1: calcium voltage-gated channel auxiliary subunit Alpha2Delta1, calcium channel; EpCAM: epithelial cell adhesion molecule, single-trans-membrane cell surface adhesion molecule; CD133: prominin 1, pentaspan transmembrane molecule; CD24, CD90: GPI-anchored cell surface molecules; CD44: single-trans-membrane cell surface molecule with multiple functions, including cell–matrix and cell–cell interactions. mTOR: mammalian target of rapamycin. Mdm2: murine double minute 2. MAPK: mitogen activated protein kinases. ERK: extracellular signal-regulated kinases.

CSCs have been characterized in HCC by different approaches. Figure 1 and Table 1 provide an overview of the most well-known HCC CSC markers and their physiological functions. Since every method to isolate CSCs relies on specific (and sometimes few) properties or individual methodological approaches, one should not consider the identified cell populations as pure, but rather as subpopulations enriched in CSCs. It is likely that the different approaches also identify varying CSC subpopulations, so comparing the results of different approaches has to be done with great caution.

A side population (SP) of cells can be isolated by flow cytometry based on their ability to efflux Hoechst dyes. This indicates their ABC-transporter activity, which is mediated by ABCG2, ABCG5 and MDR1 [150]. This side population was first identified in two out of four tested HCC cell lines [151], and sorting for these cells revealed that in xenotransplantation models, 1000 SP cells generated tumors, while 1 × 10^6^ non-SP cells were unable to do so. Furthermore, tumors derived from SP cells differentiated into SP and non-SP cells and showed increased expression of stemness-associated genes. Similar to the results in cell lines, a corresponding SP was identified in primary HCCs [152], establishing the side population as a putative CSC population in HCC and linking a CSC phenotype to drug resistance (Table 2).

CD24 was identified as a CSC-associated cell surface marker in HCC by gene expression profiling. CD24+ cells were enriched by cisplatin treatment in xenotransplanted mice and increased in tumorigenicity upon serial transplantation. Interestingly, CD24 expression was associated with worse clinical outcomes and is linked to stemness characteristics. Furthermore, CD24 expression is essential for metastasis, differentiation and self-renewal in HCC cell lines. Via phosphorylation of STAT3, CD24 induces NANOG, a key factor for self-renewal in embryonic stem cells and during tumor development [143,144], thus enabling HCC cell self-renewal [145]. In addition to increased cisplatin resistance, CD24 expression correlates with sorafenib-resistance and clinical outcome in HCC patients. This was shown to be due to the activation of autophagy, and was partially reversible upon autophagy inhibition [159]. CD24 expression in HCC has also been associated with iNOS-induced TACE/ADAM17-dependent activation of Notch1, which is linked to tumor progression and stemness [160].

CD24 expression in HCC largely overlaps with the expression of several other important CSC cell surface markers; namely, CD133, CD44 and EpCAM. CD133 has been detected in HCC cell lines and primary HCC tissue, and isolated CD133+ cells show high tumorigenicity and clonogenicity compared to CD133− cells [161]. In the human hepatoma cell line Huh-7, CD133+ cells formed tumors in SCID mice more efficiently than their CD133− counterparts, and the CD133+ population expressed fewer markers of mature hepatocytes such as glutamine synthetase and cytochrome P450 3A4 [162]. Others have found that CD133 expression is linked to a broader differentiation capacity; enhanced self-renewal and colony forming ability; and increased expression of stemness genes [38,146]. On a functional level, CD133 has been found to be associated with neurotensin-induced IL-8 and CXCL1 pathway activation, leading to enhanced MAPK pathway activity, which in turn results in increased tumorigenicity, angiogenesis and self-renewal. Similar to most CSC markers, CD133 expression has been associated with worse outcomes in HCC [163]. In an attempt to target CD133 therapeutically, Smith et al. used an anti-CD133-antibody (AC133) conjugated to the cytotoxic drug monomethyl auristatin F (MMAF) to effectively target CD133+ HCC cells. Importantly, this functionalized antibody effectively delayed the growth of Hep3B-derived tumors in SCID mice [164]. Interestingly, the NIH has listed a CD133-CART (chimeric antigen receptor T-cell) phase-I study for several malignant tumors, including liver cancer (NCT02541370), underscoring the potential usefulness of strategies targeting CSCs.

The CSC-associated surface marker CD44 has many physiological functions, including binding to hyaluronic acid, osteopontin, fibronectin and collagen [165], and it is a co-stimulatory protein of the EGF receptor and c-Met. CD44 is upregulated on TICs and proliferating cells in HCC [166], and has been shown to be essential for EMT in breast cancer [167]. CD44−/− mice were resistant to HCC induction using the hepatic procarcinogen diethylnitrosamine, and CD44 on murine hepatocytes is induced by carcinogen exposure and suppresses p53-mediated genomic surveillance via AKT and Mdm2 [148]. The upregulation of CD44 was due to IL-6/STAT3 signaling, the latter of which has already been described as a mechanism of HCC induction [168] and CSC maintenance [169]. In Huh7 cells, the CD44 standard isoform (CD44s) was shown to be required for tumorigenicity and drug resistance. Its loss also downregulated the expression of other CSC markers, including CD133 and EpCAM; impaired the antioxidative capacity of Huh7 cells via downregulation of glutathione peroxidase 1; and reduced NOTCH3 and its target genes. Again, like other CSC markers, CD44 expression is associated with poor patient prognosis [170]. While CD44 has been thoroughly evaluated as a therapeutic target, its translation to the clinic has been minimal, likely due to several detectable CD44 isoforms that are generated through alternative splicing [171]. Interestingly, since an activation of xCT (a part of the glutathione redox cycle responsible for L-glutamate transport) was shown to be mediated by CD44 on tumor cells [172], an F^18^-labeled L-glutamate analogue is currently in clinical evaluation as a tracer for PET imaging (NCT02379377).

EpCAM, a transmembrane protein with homotypic cell–cell adhesion function, is widely expressed on many epithelial cancer cells. HCCs can be subdivided into EpCAM+ tumors with a progenitor-like gene expression pattern and EpCAM- tumors with a more mature phenotype. This was demonstrated in microarray analyses in a large cohort of HCC tumors, where stemness-associated genes, such as c-Kit and Wnt/β-catenin, were upregulated in EpCAM+ HCCs [126]. EpCAM expression in HCC depends directly on Wnt/β-catenin activity, and the downstream transcription factor Tcf-4 was shown to directly bind the EpCAM promotor [173]. Additional efforts have led to the discovery that ZFX, a zinc-finger transcription factor known to promote stemness in human stem cells, augments β-catenin nuclear translocation and Wnt activation in HCC CSCs, enhancing their stemness [174]. Furthermore, EpCAM represents an important therapeutic target, with EpCAM-targeting antibodies being assessed in several clinical trials. EpCAM is a tempting target due to its strong expression on tumor cells and on CSCs. Catumaxomab, an FDA-approved chimeric antibody that binds to antigens CD3 and EpCAM, is used in the treatment of malignant ascites and has shown promising effects in malignant pleural effusion [175]. Furthermore, CART-cells with specificity against EpCAM are used in clinical trials (e.g., NCT03013712). Likewise, activated cytokine induced killer (CIK) cells with EpCAM specificity are currently in development (NCT03146637).

CD90 is a glycoprotein of the immunoglobulin family of surface proteins that was linked to CSCs in HCC due to their ability to recreate tumors in serial transplantation experiments after isolation from the blood of HCC patients [30,176]. Interestingly, compared to EpCAM, CD90 seems to be expressed on different CSC populations. Yamashita T et al. demonstrated that while EpCAM appears to enrich for CSCs with a more epithelial gene expression profile, CD90+ HCC cells were found to be more mesenchymal. Accordingly, EpCAM+ cells revealed local tumorigenicity, while CD90+ cells efficiently formed lung metastases upon transplantation in mice. However, in co-cultures, these subpopulations positively interacted with CD90+ cells enhancing the motility of EpCAM+ cells in vitro through TGF-β signaling. The authors also showed high c-Kit expression in metastatic CD90+ cells, suggesting therapeutic potential for imatinib against CD90+ CSCs [177]. CyclinD1-dependent SMAD activation also appears to be essential for the maintenance and chemosensitivity of CD90+ CSCs in HCC. Specifically, Xia et al. demonstrated that >50% of xenotransplanted HCCs could be eliminated by SMAD inhibition [178]. It is important to note, however, that the used small molecule inhibitor SB431542 is a potent inhibitor of the TGF-β/Activin/NODAL pathway that inhibits ALK5, ALK4, and ALK7 and not SMADs directly. The link between CD90 and a more metastatic CSC phenotype was further explored in a study aimed at the identification of markers for circulating tumor stem cells. The authors showed that CXCR4 is an essential contributor to CD90+ mediated distant metastasis, as only CD90+CXCR4+ HCC cells developed metastases in NOD/SCID mice, whereas CD90−CXCR4−, CD90−CXCR4+ and CD90+CXCR4− cells failed to do so [179]. The use of CXCR4 as a marker to identify metastatic CSCs is in line with previous studies in pancreatic cancer, in which we showed that CD133+/CXCR4+ CSCs have exclusive tumorigenic and metastasis-initiating capacity [9]. CD90 expression negatively impacts prognosis [180], but targeting CD90 as a means of treating HCC is still at the experimental stage and no drugs targeting CD90 are approved currently. A very interesting study using magneto-liposomes (to induce local hyperthermia via magnetic fields) loaded with Fe_3_O_4_ and anti-CD90 antibodies to target Huh7-induced HCCs in mice resulted in tumor size reduction and showed specificity against CD90+ CSCs [181]. Similarly, using the “energy restriction mimetic” OSU-CG5, Chen WC et al. were able to show reduction of the CD90+ population in fresh liver tumor samples and repression of the tumor growth established with HCC cell lines with ectopic CD90 expression [147].

CD13 can serve as a CSC marker in HCC [182]; however, it is only expressed in a semi-quiescent CSC subpopulation. CD13+ cells are predominantly in G0-phase and were found to be enriched in the tumor periphery after treatment with doxorubicin or 5-FU. Functionally, CD13 protects cells from genotoxic damage by activating reactive oxygen species (ROS) scavenging systems and by supporting CSC self-renewal. Importantly, inhibition of CD13 using ubenimex led to a strong increase in therapeutic efficacy of 5-FU treatment of HCCs in xenograft models and hampered the ability of CSC to self-renew and initiate tumors [154], indicating sensitization of CSCs to therapy. The same authors linked TGF-β-induced EMT to CD13 upregulation, promoting CSC survival by abrogating ROS production [183]. Several experimental approaches have been designed to target CD13 in HCC, most of them showing promising efficacy, especially in combination with cytotoxic agents [184,185,186,187,188,189]. The above-mentioned ubenimex is a protease inhibitor blocking CD13 activity and has been evaluated in other diseases; i.e., squamous cell lung carcinoma, lymphedema and pulmonary hypertension. It is usually well-tolerated, but while effective against CD13+ CSCs in mouse models of HCC [154], clinical trials investigating ubenimex in HCC are still lacking.

OV-6 is the epitope for anti-OV-6 antibodies derived from hybridomas generated from BALB/c mice immunized with the oval cell antigen [190]. OV-6 was shown to be expressed in ductular and hepatocytic regenerates after liver injury in humans [191] and in HCC cell lines [192], and in primary HCCs [193]. OV-6 expression has been associated with CSC traits [194], and OV-6+ HCC correlated with poorer prognosis in tissue microarrays containing samples from 208 HCC patients [195]. While studies in esophageal carcinomas have found a link between OV-6 expression and β-catenin stabilization via ATG7, a similar link still needs to be demonstrated in HCC. However, OV6+ CSCs have already been described to have highly active Wnt/β-catenin signaling in HCC [196]. 

Zhao et al. identified isoform 5 of the cell surface calcium channel α2δ1 as a putative CSC marker in HCC in 2013 [149]. When α2δ1+ cells were subcutaneously injected in NOD/SCID mice, they showed increased tumorigenic potential in comparison with α2δ1− cells. Indeed, α2δ1+ cells expressed other CSCs markers, such as CD133, CD13 or EpCAM, further supporting this subpopulation of cells to be CSCs. Tissue sample analysis showed that α2δ1 could be found in most HCC samples and in surrounding tissue, the latter correlating with pathological factors, such as cirrhosis and shorter survival. Regarding the biological role of α2δ1 overexpression in CSCs, it was demonstrated that α2δ1 regulates calcium signaling, and intracellular calcium concentration and oscillation were shown to be higher in α2δ1+ cells. α2δ1 silencing leads to suppressed ERK1/2 phosphorylation, which suggests a role for calcium flux in CSC biology. This hypothesis was supported by ERK1/2 inhibition experiments [149]. Other types of cancer also contain a subset of α2δ1+ cells (small-cell lung cancer, laryngeal squamous cell carcinoma and gastric cancer), showing not only the same stem cell-like properties [197], but also the same chemoresistant phenotype [198] and overexpression of ERK1/2 [199]. In all cases, inhibition of α2δ1 has been proposed as a new promising therapeutic target for CSCs, including HCC CSCs [197,198,199,200].

### 3.3. Hepatitis B and C Viral Infections in HCC Initiation and CSC Maintenance

While the genetic origins of HCC are still not completely known, with no one set of driver mutations predominating as with other cancers, it is clear that HCC development is tightly linked to chronic liver damage and inflammation [201]. Thus, it is not surprising that infection with HBV or HCV has been determined as one of the leading risk factors for HCC development. As with alcohol-steatohepatitis-related HCC, cirrhosis due to chronic inflammation is likely to also occur before viral hepatitis-related HCC. However, other risk factors, such as alcohol consumption, obesity or diabetes, can synergize with chronic viral hepatic infections, promoting a multifactorial evolution towards HCC [202].

The link between HBV or HCV infection and HCC raises many questions with respect to the genesis of CSCs, such as whether hepatitis viruses promote the evolution of CSCs, whether these viruses preferentially replicate in CSCs, or whether viral products/replication can regulate stemness in cancer cells. It has been reported that cells with CSC markers—such as EpCAM—expressing stem-like transcriptional factors, such as Nanog or Oct4, also express the Hepatitis-B X-protein (HBx) of HBV, in either normal or truncated form [203,204]. HBx is a 16.5 KDa viral protein that apart from playing a role in the HBV life cycle, can activate mitogenic signaling cascades, altering expression of proliferation genes via transcription factors such as NF-κB, AP-1, AP-2, c-EBP and ATF/CREB [205]. HBx, however, is not the only HBV protein related to a stem-like phenotype. The viral PreS1 protein induces CD133, CD117 and CD90 expression in cancer cells, leading to an increase in sphere formation, migration, tumorigenesis and tumor growth in nude mice [206]. Other studies have suggested that HBV-positive cells have increased Wnt pathway activity, mediated by upregulation of EpCAM after loss of PCR2 function, leading to the expression of CSCs markers and resulting in poor prognosis [207]. The chronic viral-related inflammatory environment has also been proposed to be an important factor that could increase CSC proliferation due to cytokine-induced enhancement of Oct4/Nanog [208].

HCV infection/replication has been directly related to the acquisition of a stem-like phenotype in HCC. HCV subgenomic replicon replication has been linked to the enhancement of stem-like properties. When expressed in HCC cells, HCV subgenomic RNA replication enhanced the expression of DCAMKL-1, LGR5, CD133, AFP, CK-19, Lin28 and c-Myc, all of which have been associated with CSCs in HCC. Importantly, inhibition of the replicon reversed this phenotype, indicating that HCV replication and the production of non-structural HCV proteins can promote a stem-like state [209]. The latter are also involved in the acquisition of a CSC phenotype (as in HBV). Toll-like receptor 4, for example, is induced by the expression of the NS5A HCV protein in hepatocytes, and this induction correlates with the upregulation of Nanog and CD133 [210]. Finally, sphere formation assays with HCV-infected and non-infected HCC cell lines showed the increased sphere formation capacity of infected cells with an upregulation of stemness markers such as CD133, c-Kit, CD105, Sox2 and CD45 [211]. Moreover, microenvironmental factors and signaling cascades can influence HCV replication, and consequently, its link to promoting HCC CSCs. Shirasaki et al. showed that the osteopontin-CD44 axis in EpCAM+/CD44+ CSCs is important for mediating higher HCV replication in these cells through STAT1 reduction. The authors observed a significant difference compared to the EpCAM/CD44 non-CSC population, suggesting that HCV can replicate to higher levels in CSCs. As detailed above, this higher replication and/or increased production of viral proteins can induce or maintain the CSC state [212].

While the precise mechanism(s) behind how HBV and HCV promote the genesis and maintenance of CSCs is still not yet entirely understood, insights gained from the above-mentioned and future studies could ultimately be used to control hepatitis virus replication and simultaneously eliminate CSCs in HCC.

### 3.4. The CSC Niche in HCC: Ectopic Lymphoid Structures and Hepatic Stellate Cells

As detailed above, CSCs need a supportive microenvironment for their maintenance and survival. This niche generates a protective and growth-promoting milieu via cellular players, the extracellular matrix (ECM) and soluble factors. The niche is of special interest, since therapeutic interventions could potentially affect the CSC pool either directly or indirectly by elimination of the protective environment. The niche is thought to protect CSCs from chemotherapy, prevent apoptosis and maintain stemness. In the case of HCC, a structure called the “ectopic lymphoid structure (ELS)” or “tertiary lymphoid tissue” has been recognized to represent a putative microenvironment that promotes CSC growth and even the “education” of TICs into fully self-sufficient CSCs [213]. Because ELSs are associated with a more favorable prognosis in some malignancies, including carcinomas of the lung, skin, colon and breast [214,215,216], they were thought to be the anatomic correlate of an anti-cancer immune response. In breast cancer, ELS mainly contains follicular helper T cells; Th1, Th2 and Th17 effector memory cells; and regulatory T cells [216]. On the contrary, in HCC, anatomical detection of these structures was associated with an increased risk for late recurrence, and using a molecular detection method based on gene expression profiling, ELSs were associated with poor overall survival. Using liver specific expression of a constitutively active IKK-β variant in mice, activated hepatic NFκB signaling induced the formation of ELS after 7 months and of HCC after 20 months in 100% of the mice analyzed in the study. Interestingly, lymphocytes were essential for tumor formation, since a cross with a RAG^−/−^ mouse with constitutive IKK-β activation resulted in a reversal of the HCC phenotype [213]. This observation is in line with the consensus that HCCs emerge in livers suffering from (non-)alcoholic steatohepatitis or viral hepatitis. Importantly, Svinarenko et al. were recently able to reproduce this signaling link in vivo using a conditional liver-specific IKK-β activation model with p53 loss [217].

Another characteristic of the CSC niche in HCC is the presence of a stromal cell population known as hepatic stellate cells (HSCs). These cells exhibit a myofibroblast-like phenotype in conditions of chronic liver injury. In cancer, these cells share many of the same functions as CAFs at the level of the CSC niche. It has been reported that HSCs secrete HGF or IL-6 that induce the expression of stemness markers in CSCs, and this increased expression is mediated through STAT3 signaling [218,219]. The investigation of the crosstalk between HSCs and CSCs resulted in the identification of the transcription factor Forkhead Box M1 (FOXM1) as a main player in HSC activation and maintenance of stemness in CSCs in vitro. FOXM1 inhibition in co-cultures of these two populations disrupted the crosstalk, whereas the overexpression of FOXM1 reversed the effects of the inhibition [220]. These lines of research highlight additional potential targets to interrupt the communication between HSCs and cancer (stem) cells, with the ultimate goal of targeting the CSC via multiple approaches.

## 4. Discussion

In the sections above, we review the current knowledge about CSCs in general and in the more specific context of HCC. Based on the CSC model, only by targeting and eliminating CSCs along with the more differentiated cells of the bulk tumor will we be able to achieve long-term curative responses and prevent metastatic spread. At the same time, CSCs display highly effective mechanisms of therapy resistance, such as the expression of drug efflux pumps, (semi-)quiescence and high ROS scavenging. Thus, it is unlikely that CSCs will be effectively targeted with classical cytostatic drugs. Furthermore, most HCC patients suffer from side effects associated with therapy, since HCC typically arises in severely damaged, cirrhotic livers. These patients suffer from chronic anemia and thrombocytopenia; are prone to infections; and often present with highly reduced (and therefore therapy-limiting) liver function and subsequently reduced dosage tolerance. One possible approach to reduce toxicity would be to utilize the inherent properties of CSCs. For example, inhibiting efflux pumps in CSCs could enhance their susceptibility to cytostatic drugs, reducing the necessary dose and thus increasing drug tolerance in the patient. For example, the calcium channel blocker verapamil showed efficacy in vitro, sensitizing SP cells to gemcitabine in pancreatic cancer [221], and has shown promising results in HCC [222,223]. ABC transporter inhibitors such as β-caryophyllene oxide have been shown to sensitize HCC cells to sorafenib by favoring its intracellular accumulation [224], but these compounds are still in development and their clinical utility is still unknown. Indeed, even third-generation inhibitors of p-glycoprotein drug transporters have not shown clinical efficacy [225]. Targeting CSC-specific surface markers or stemness-associated pathways for cancer therapy has led to several clinical trials that are currently ongoing to test highly specific molecules or antibodies that target CSCs. However, a key obstacle with these strategies is the expression of these markers or pathways on/in other stem cells, such as a hematopoietic stem cells or adult stem cells; another is the subsequent adverse effects of a targeted CSC therapy. In summary, the recognition of CSCs as a target sheds new light on potential therapeutic approaches in HCC, even though effective treatments against these highly aggressive cells still need to be developed.

## 5. Conclusions

Several lines of evidence point to the CSC as a key therapeutic target in HCC, as in many other tumors. Thus, this population must be successfully eliminated in order to prevent metastatic spread and tumor relapse, but due to their high therapy resistance and high cellular plasticity, effective means still have to be found to specifically identify these cells and to adequately target them, especially in HCC. To conclude on a promising note, the recent IMbrave150 trial showed impressive therapeutic effects of a combined anti-VEGF/anti-PD-L1 targeted therapy in HCC. In this trial, overall survival and progression free survival of HCC patients improved significantly and relevantly compared to sorafenib in first-line treatment, making this trial the first successful first-line trial in HCC since more than a decade (NCT03434379). Targeting CSCs could in part account for this positive outcome, since in many tumor types, a selective upregulation of PD-L1 has been observed on CSCs [226,227,228,229,230], enabling them to even induce apoptosis of T-cells in vitro. Furthermore, PD-L1 expression seems to be linked to EMT via ZEB-1 [231], which is highly expressed in HCC [232], closing the circle of CSC, EMT, therapy resistance and immune evasion. Several other mechanisms for immune evasion have been demonstrated in CSC, but available data are still sparse and in part conflicting [233]. MHC-I expression was lower in CSCs of colorectal cancer and glioblastoma patients [226,234,235]. Likewise, defects in the antigen processing machinery of CSC were observed [235]. In conclusion, these findings point to mechanisms of immune evasion that are highly active on CSC, and therefore, CSC depletion could have contributed to the success of the IMbrave150 trial. On the other hand, the single agent anti-PD-1 trial CheckMate 459 missed its primary endpoint of overall survival, which points to additional effects of the anti-VEGF agent and its possible interference with CSCs by mechanisms described elsewhere in this article.

## Figures and Tables

**Table 1:** Surface molecules linked to cancer stem cell (CSC) traits in HCC and their putative oncogenic and stemness supporting functions (Figure 1).

**MDR Proteins**	Upregulation in HCC-CSC and contribute to drug resistance by active outward transport of drugs [31]
**CD24**	Upregulation in HCC CSC leads to Nanog-upregulation and therefore stemness-conservation [143,144,145]
**CD133**	Activates autocrine signals ultimately leading to pro-oncogenic MAPK signaling [38,146]
**CD90**	Activates AMPK and its downstream target mTOR [147]
**CD44**	Mdm2 Activation [148]
**EpCAM**	Induced by β-catenin signaling [126]
**α2δ1**	Subunit of voltage-gated calcium channel complex, ERK1/2 activation [149]

**Table 2:** Major mechanisms of resistance of CSCs to therapy.

Mechanisms of Resistance to THERAPY of CSCs
Dormancy: G_0_-cell cycle arrest with high resistance to genotoxic agents [153]
Increased DNA-Repair and reduced apoptotic response to DNA damage [154,155,156]
Active efflux-pumping of antineoplastic agents via MDR proteins [157]
Anti-apoptotic tumor microenvironment: The Cancer Stem Cell Niche [158]
